# Surgical management of laryngeal bilateral abductor palsy: comparative study between carbon dioxide and diode lasers

**DOI:** 10.1007/s10103-022-03589-x

**Published:** 2022-06-14

**Authors:** Ahmed El-Sobki, Mohamed E. El-Deeb, Noha Ahmed El-Kholy, Fedaey R. Habaza, Mahmoud Ahmed Shawky, Mahmoud Elsaid Ibrahim Alsobky

**Affiliations:** 1 Otorhinolaryngology Department, Faculty of Medicine, Mansoura University, Mansoura, Egypt; 2 Otorhinolaryngology Department, Faculty of Medicine, Kafrelsheikh University, Kafrelsheikh, 33155 Egypt; 3 Otorhinolaryngology Department, Faculty of Medicine, Al-Azhar University, Damietta, Egypt

**Keywords:** Diode laser, CO_2_ laser, Bilateral vocal fold paralysis, Glottis

## Abstract

This study aims to compare the results of both CO_2_ laser and diode laser combined arytenoidectomy with posterior cordectomy in managing patients with bilateral vocal fold paralysis. A prospective study involved 80 bilateral vocal cord immobility patients in adduction. They are divided into two groups according to the laser used, whether CO_2_ (with a wavelength of 10.6 µm) or diode (with a wavelength of 980 nm). We used mMRC (Modified Medical Research Council) dyspnea scale to assess dyspnea in our patients, while the voice was evaluated by both maximum phonation time and the voice handicap index. Quantitative variables were described using means and standard deviations, while categorical variables were described using frequencies and were compared using the chi-square test, Fisher exact test, and Monte Carlo test. There was a statistically non-significant difference between the studied groups regarding mMRC dyspnea scale and Voice Handicap Index preoperatively and postoperatively. There is a statistically significant difference between the two groups regarding maximum phonation time postoperatively (significantly higher in the CO_2_ laser group) (*p* < 0.001). The CO_2_ laser and diode laser could be used safely for the management of bilateral vocal cord paralysis. The CO_2_ laser maintains better voice parameters and less postoperative pain, while the diode laser gives less operative time, lower cost, and simplicity of use.

## Introduction

Bilateral vocal fold immobility (BVFI) is caused by thyroid surgeries, neck trauma, neurological disorders, laryngeal malignancies, and others [1]. It is usually presented by dyspnea and noisy inspiratory breathing with some voice changes [2].

Many surgical procedures have been used to treat respiratory distress secondary to bilateral vocal fold paralysis (BVFP). The success of these techniques depends on their ability to balance phonation, airway, and swallowing. Posterior cordectomy with arytenoidectomy is considered a well-established method of treating BVFI [3, 4].

This procedure was reported to be done by various instruments, including cold instrumentation, diathermy, laser, and coblation, each with its advantages and disadvantages [5]. The CO_2_ laser wavelength has a very high affinity for water, resulting in rapid soft tissue removal and hemostasis with a very shallow penetration depth [6]. In contrast, diode wavelengths are absorbed primarily by tissue pigment (melanin) and hemoglobin [7].

To the best of our knowledge, there are no comparative studies evaluating outcomes of CO_2_ and diode laser cordectomies. This study aims to compare the results of CO_2_ laser and diode laser combined arytenoidectomy and posterior cordectomy in the treatment of the challenging cases of bilateral abductor paralysis. We demonstrate if there is a difference in voice and dyspnea parameters according to the laser used, whether CO_2_ or diode, or not.

## Materials and methods

**Fig. 1:**
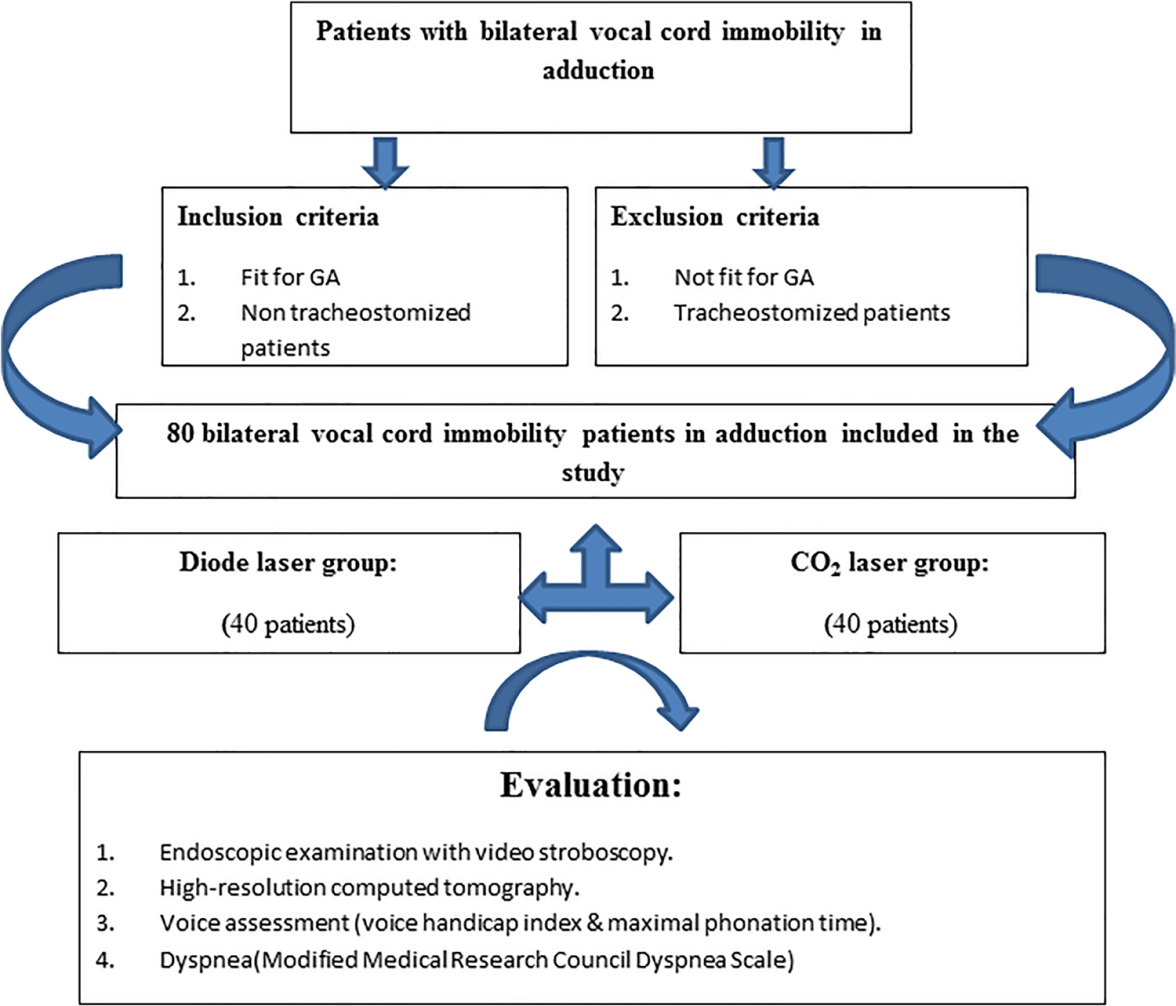
Diagrammatic summary presentation of our patients

This prospective study involves eighty patients diagnosed with bilateral abductor paralysis during the period from May 2016 to April 2021. According to the laser used, they are divided into two groups, whether CO_2_ or diode (FIG. 1). The university ethical committee approved this study and informed written consent was obtained from all patients.

### Sample size calculation

The mean and standard deviation (SD) for Voice Handicap Index-Physical domain (VHI-P) were 11 ± 4.8 preoperative and 14.88 ± 7.22 postoperative [8] using OpenEPI software; the sample size was calculated to be 40 patients within each group (1:1 distribution) at confidence level 95% and power of study 80%.

### Preoperative evaluation

The etiology and duration of paralysis were documented by a thorough history taking. Endoscopic examination of each patient was done together with video stroboscopy: a 70° rigid endoscope and a Kay Digital Strobe 9200 (Kay Elemetrics Co., Pine Brook, NJ, USA) to determine the glottic chink. A high-resolution computed tomography (CT) scan of the neck and chest was performed for all patients to rule out organic lesions.

Voice assessment was done both preoperatively and 3 months postoperatively using both Voice Handicap Index (VHI), the Arabic version [9], and maximum phonation time (MPT) [10]. Dyspnea was assessed by mMRC (Modified Medical Research Council) dyspnea scale [11].

We usually operated upon the cord with less residual mobility or the thicker cord of which targeting would get bigger space. If there was no difference, we operated on the left cord because it was much easier for the right-handed surgeon.

### Operative technique

The operative microscope used was OPMI 1-FC, Carl Zeiss Meditec, Jena, Germany, with an objective length of 400 mm.

#### CO_2_ laser group

We used the C.LAS machine (C.LAS, A.R.C., Nuremberg, Germany) connected to the aforementioned microscope and a micromanipulator (ACCU-beam, TTI medical, San Ramon, California, USA) with a wavelength of 10.6 µm. Glottis exposure was achieved using the largest possible laryngoscope for comfortable manipulation. The procedure starts with an incision of the selected vocal cord transversely. This incision site is a few millimeters in front of the tip of the arytenoid’s vocal process. The smallest and most focused beam was used in the continuous super pulse mode 5 W setting. The incision was continued laterally to cut the ventricular fold and manipulated posteromedially to remove part of the arytenoid body and the whole vocal process. When starting to ablate cartilage, the machine’s setting was changed to 10 W continuous mode.

#### Diode laser group

The laser used was the ARC diode laser (Fox, A.R.C., Nuremberg, Germany). We used a wavelength of 980 nm in our work. A wedge of the posterior one-third of the vocal cord is excised from the free border of the membranous cord, anterior to the vocal process, extending laterally till the false vocal cord, 2–3 mm of the ventricular fold was incised. Then the cartilage of the arytenoid body posterior to the vocal process is removed with the laser. The arytenoid’s 1–2-mm posterior shell, the inter-arytenoid area, and posterior commissure should be protected. Dealing with the vocal cord was done using 6 W in continuous mode, while 10 W in continuous mode was selected for the arytenoid cartilage.

### Postoperative care and assessment

Semisolid feeding was started 6 h postoperatively, while clear fluids were allowed after 24 h. Antireflux treatment (Antropral, Sandoz a Novartis division, Cairo, Egypt) was prescribed for 3 months, and speech therapy was started after 3 weeks. Patients were followed-up at the following intervals: after a week, 2 weeks, a month, 3 months, and 6 months.

### Statistical analysis

Data analysis was performed using SPSS (Statistical Package for the Social Sciences) version 20 (IBM SPSS Statistics for Windows, Armonk, NY: IBM Corp., Chicago, Illinois, USA). Quantitative variables were described using means and standard deviations, while categorical variables were described using frequencies and were compared using the chi-square test, Fisher exact test, and Monte Carlo test when appropriate. Kolmogorov–Smirnov (distribution-type) and Levene (homogeneity of variances) tests were used to verify assumptions for use in parametric tests. To compare continuous variables between two groups, an independent sample *t* test when data was normally distributed and Mann Whitney test when data was not normally distributed were used. Paired sample *t* test and Wilcoxon signed-rank test were used to compare the change in continuous parametric and non-parametric variables, respectively, within the same group over two time points. Statistical significance was set at *p* < 0.05. The sample size was calculated to be 40 patients within each group (1:1 distribution) at a confidence level of 95% and power of study of 80%.

## Results

Our prospective study included eighty patients divided into two groups according to the type of laser used, whether CO_2_ or diode laser. No statistically significant difference was detected between the studied groups regarding basic data such as age, gender, or cause of vocal cord injury **(Table ****1****)**. Also, there was no statistically significant difference regarding the mMRC dyspnea scale and Voice Handicap Index preoperatively or postoperatively **(Table ****2****)**.

**Table 1:** Comparison between the studied groups regarding demographic data

**Parameter**	**Groups**			**Test**
	**Diode laser group**	**CO**_**2**_** laser group**	**χ**^**2**^	***p***
	***N* = 40 (%)**	***N* = 40 (%)**		
**Gender:**				
Female Male	26 (65.0) 14 (35.0)	24 (60.0) 16 (40.0)	0.213	0.644
**Age (year):**				
Mean ± SD Range	48.85 ± 6.81 36–66	47.88 ± 7.96 29–63	0.589	0.558
**Cause:**				
Esophageal surgery Intubation trauma Open neck trauma Thyroidectomy Tracheal surgery Viral neuritis	2 (5.0) 3 (7.5) 3 (7.5) 26 (65.0) 2 (5.0) 4 (10.0)	4 (10.0) 3 (7.5) 2 (5.0) 25 (62.5) 3 (7.5) 3 (7.5)	MC	0.977

*t* independent sample *t* test, **χ**.^**2**^Chi square test, *MC* Monte Carlo test

**Table 2:** Comparison between the studied groups regarding disease-specific measures preoperative and postoperative

**Parameter**	**Groups**			**Test**
	**Diode laser group**	**Co2 laser group**	**t/Z**	**p**
	**Mean ± SD**	**Mean ± SD**		
**Voice handicap index (VHI):**				
Preoperative Postoperative	37.38 ± 6.32 41.73 ± 6.63	37.43 ± 6.23 38.9 ± 6.13	− 0.107 1.979	0.915 0.051
**P (pt)**	< 0.001*	< 0.001*		
**Maximum phonation time (MPT):**				
Preoperative Postoperative	8.0 ± 0.99 6.4 ± 1.19	7.9 ± 0.98 7.55 ± 0.88	0.454 − 4.912	0.651 < 0.001**
**P (pt)**	< 0.001*	< 0.001*		
**mMRC dyspnea scale:**				
Preoperative Postoperative	3 (2–4) 1 (0–2)	3 (2–4) 1 (0–2)	− 1.555 − 1.407	0.12 0.16
**P (Wx)**	< 0.001*	< 0.001*		

^*^p < 0.05 is statistically significant. *t* independent sample *t* test, *Z* Mann Whitney test, *pt* paired sample *t*-test, *Wx* Wilcoxon signed-rank test

Although there was a statistically non-significant difference between the studied groups regarding MPT preoperatively, there was a statistically significant difference postoperatively, significantly higher in the CO_2_ laser group (*p* < 0.001). A significant improvement in each of these parameters postoperatively within each group was noted (*p* < 0.001) **(Table ****2****)**.

There was a statistically significant difference between the studied groups regarding percent change in VHI and MPT (*p* < 0.001) (percent change was significantly higher in the diode laser group). Four patients (10%) in the diode laser group versus twenty-six patients (65%) within the CO_2_ laser group had no change in MPT postoperatively **(Table ****3****)**.

**Table 3:** Comparison between the studied groups regarding percent change disease-specific measures preoperative and postoperative

**Parameter**	**Groups**			**Test**
	**Diode laser group**	**Co2 laser group**	**Z/χ**^**2**^	**p**
	**Mean ± SD**	**Mean ± SD**		
**Voice handicap index (VHI)**				
Median Range	10.96% 2.22– 36.67%	3.45% 0–11.11%	− 5.903	< 0.001*
**No change**	0 (0)	5 (12.5%)	Fisher	0.055
**Maximum phonation time (MPT)**				
Median Range	− 20% − 50–0%	0 − 14.29–0%	− 6.111	< 0.001*
**No change**	4 (10%)	26 (65%)	25.813	< 0.001*
**mMRC dyspnea scale**				
Median Range	− 66.67% − 100, − 33.3%	− 66.67% − 100, − 33.3%	− 0.787	0.431

^*^ *p* < 0.05 is statistically significant. *Z* Mann Whitney test

All patients within the diode laser group had a postoperative change in VHI. There was a statistically non-significant difference between the studied groups regarding percent change in the mMRC dyspnea scale **(Table ****3****)**.

There was a statistically significant difference between the studied groups regarding operation time (*p* < 0.001) (longer in the CO_2_ laser group). There was a statistically significant difference between the studied groups regarding VAS pain score postoperatively (*p* < 0.001) (significantly higher in the diode laser group) **(Table ****4****).**

**Table 4:** Comparison between the studied groups regarding operation time and VAS pain score

**Parameter**	**Groups**			**Test**
	**Diode laser group**	**CO**_**2**_** laser group**	**t**	***p***
	*N* = 40 (%)	*N* = 40 (%)		
**Operation time**				
Mean ± SD Range	15.93 ± 1.654 12–18	25.48 ± 2.05 19–30	-23.54	< 0.001*
**VAS pain score**				
Mean ± SD Range	6.23 ± 0.86 4–8	3.63 ± 0.9 2–6	13.219	< 0.001*

^*^p < 0.05 is statistically significant, *t* independent sample *t* test

Concerning swallowing, we had five patients with temporary aspiration (three in the diode laser group and two in the CO_2_ group). Patients were managed conservatively by asking them to tilt the neck to the non-operated side while drinking, with subsequent improvement within 2 weeks. Three patients suffered from postoperative granuloma (two in the diode laser group and one in the CO_2_ group). None of our patients had postoperative bleeding or stenosis.

## Discussion

The surgical management of bilateral abductor paralysis should be a balance between getting an adequate airway together with the preservation of the proper phonatory quality [12].

Lasers have been used for years in laryngeal surgeries [13]. The properties of having a precise cut with good water absorption have made CO_2_ laser the most preferred type for transoral laser microsurgery. However, its difficult transportation and high cost hindered its generalized use [14].

On the other hand, the diode laser is relatively inexpensive and easy to manipulate and transport with good hemostatic properties due to its good absorption by hemoglobin, but it is less absorbable by water than the CO_2_ laser [15, 16].

In the present study, posterior cordectomy with partial arytenoidectomy was performed in 80 patients. These patients are divided into two groups: 40 patients with the aid of a diode laser and 40 patients with a CO_2_ laser.

Gender distribution in our study was 26 females and 14 males in group 1 (diode laser group) and 24 females and 16 males in group 2 (CO_2_ laser group). The mean age in our study population was 48.85 ± 6.81 SD in group 1 and 47.88 ± 7.96 SD in group 2. The most common etiology of BVFP in our 80 case study was post thyroidectomy, followed by viral neuritis, intubation trauma, open neck trauma, and esophageal and tracheal surgery. There was a statistically non-significant difference between the studied groups regarding gender, age, or cause of vocal cord injury, in accordance with Khalil et al. [17], who conducted laser posterior cordotomy on 18 patients with bilateral vocal fold abductor paralysis, 10 females (55.5%) and 8 males (45.5%). Their ages ranged between 32 and 64 years. The current study’s most common cause for bilateral abductor paralysis is a thyroidectomy. Also, nearly the same demographic data and etiological factors were obtained by Manolopoulos et al. [18], who conducted a study of CO_2_ and KTP-532 laser cordectomy for bilateral abductor paralysis.

In this study, posterior cordectomy with partial arytenoidectomy was performed according to the method described by Maurizi et al. [19], who believed that arytenoidectomy (subtotal or total) should be done to achieve satisfying respiratory outcomes and decrease the chance of tissue shrinkage. Eckel et al. [20] found that voice outcomes are not predictable with either cordectomy or arytenoidectomy. Also, Bosley et al. [21] found that both medial arytenoidectomy and transverse cordotomy can enlarge the laryngeal airway with a minimal negative impact on phonatory and swallowing function.

In comparing the 2 groups regarding the VHI and mMRC dyspnea scale, we found a statistically non-significant difference between the studied groups regarding the mMRC dyspnea scale and VHI preoperatively or postoperatively **(Table ****2****)**.

A statistically significant decrease in mMRC dyspnea scale postoperatively was achieved in both groups, which is our target (*p* < 0.001) **(Table ****2****)**. These results are nearly similar to those obtained by Asik et al. [22]. In their study, patients improved after posterior cordotomy according to mMRC dyspnea scale results from 2.9 ± 0.7 before surgery to 0.9 ± 0.5 after the surgery, revealing a statistically significant improvement (*p* = 0.003). Jackowska et al. [23] found that 91% of non-tracheostomized patients who underwent posterior cordectomy reached respiratory comfort.

Assessment of voice with VHI comparison between the 2 groups resulted in a statistically significant difference between the studied groups regarding percent change in VHI. Percent change was significantly higher in the diode laser group.

Going with El-Sobki et al. [24], who used the VHI to assess the patient’s self-evaluation of voice handicap in a diode laser cordectomy with arytenoidectomy. They found no significant difference between the score preoperatively and postoperatively.

Khalil et al. [17] accomplished CO_2_ laser posterior cordotomy in 18 patients, assessing voice outcome after the operation with the Voice Handicap Index (VHI after translation into the Arabic language) after 3 months and 1 year of the operation. In parallel to our study results, all the patients were satisfied with their voices after the operation. Lawson et al. [3] reported a good voice quality objectively after 15.2 months after the posterior cordectomy procedure.

Also, in harmony with our results, Karasu et al. [25] compare the effects on the voice of endolaryngeal microsurgery (EMS) with cold instruments and a new method, ‘‘diode laser,’’ for vocal fold polyps. There was a significant difference in VHI between the score obtained preoperatively compared with at follow-up for each group. There was no significant difference in VHI score between the two groups postoperatively. This finding was interpreted as a significant improvement in hoarseness for the two groups.

Their satisfaction may be explained by proper counseling of patients about the potential worsening of vocal performance after surgery; they expect their voices to worsen. This is likely to influence the responses on the perceptual scale [22].

Regarding MPT preoperatively, there is a statistically non-significant difference between the studied groups' MPT preoperatively (*p* = 0.651) **(Table ****2****)**. On the other hand, there was a statistically significant difference between the studied groups regarding MPT postoperatively (significantly higher in the CO_2_ laser group) (*p* < 0.001) **(Table ****2****)**.

Parallel to El-Sobki et al. [24], the MPT significantly decreased from 8.04 ± 0.978 preoperatively to 6.92 ± 0.997 postoperatively (*p* < 0.001). The MPT averaged 6.57 s with a median of 6 s as opposed to 10 s postoperative in Plouin-Gaudon et al. [26]. The MPT tends to decrease after glottal widening, as expected, because of creating a posterior gap [26].

Hillel et al. [27] concluded from their study that posterior cordotomy with medial arytenoidectomy offered patients improved or unchanged voice quality of life, despite the decrease in the overall voice severity perceived by professionals.

The explanation of the better voice outcome with CO_2_ laser can be that its minimal collateral damage leaves the vocal cord’s residual anterior portion better vibrating. The deeper penetration of the diode laser could make mucosal waves less efficient.

Regarding complications, five patients had temporary aspiration, which improved conservatively by asking them to tilt the neck to the non-operated side while drinking. Postoperative granuloma occurred in three patients at the operative site. This was managed by surgical removal with intralesional corticosteroid injection. None of our patients had postoperative bleeding or stenosis. Plouin-Gaudon et al. [26] found that aspiration episodes are the most frequent immediate postoperative complication, which usually resolves spontaneously after a few days to weeks. Bizakis et al. [28] reported initial aspiration in 22.2% of patients who underwent combined posterior cordectomy with total arytenoidectomy, but this resolved in a few days without the need for any further treatment.

In Al-Fattah et al.’s [29] study of partial arytenoidectomy, postoperative obstructive granulation tissue was addressed in only 4.4% of patients, while Dursun and Gökcan [30] reported granulation tissue formation in 27.3% of patients after CO_2_ bilateral cordotomy.

Regarding our operative time, we used 12–18 min in group 1 to accomplish posterior cordectomy with partial arytenoidectomy compared to group 2, in which we used 19–30 min in the same procedure. There was a statistically significant difference between the studied groups regarding operation time (longer in the CO_2_ laser group). That was nearly the same operative time consumed by Remacle et al. [31] doing subtotal carbon dioxide (CO_2_) laser arytenoidectomy for endoscopic treatment of bilateral immobility of the vocal folds in adduction in which the surgical procedure lasts 25–30 min.

The overall advantage of our study is that it is a prospective study and comparison between different types of laser in the management of bilateral abductor paralysis. The limitation of our study is that the follow-up period is still short, and a larger sample of patients is needed. In the future, objective voice assessment, including measuring variations in fundamental frequency, Jitter %, Shimmer %, and Harmonics-to-Noise Ratio (HNR), can be used. Peak inspiratory flow (PIF) can also be used to objectively evaluate respiratory function.

## Conclusion

The CO_2_ laser and diode laser could be used safely for the management of bilateral abductor paralysis. The CO_2_ laser maintains better voice parameters and less postoperative pain, while the diode laser gives less operative time, lower cost, and simplicity of use.
